# Structures of N-terminally processed KRAS provide insight into the role of N-acetylation

**DOI:** 10.1038/s41598-019-46846-w

**Published:** 2019-07-19

**Authors:** Srisathiyanarayanan Dharmaiah, Timothy H. Tran, Simon Messing, Constance Agamasu, William K. Gillette, Wupeng Yan, Timothy Waybright, Patrick Alexander, Dominic Esposito, Dwight V. Nissley, Frank McCormick, Andrew G. Stephen, Dhirendra K. Simanshu

**Affiliations:** 1 NCI RAS Initiative, Cancer Research Technology Program, Frederick National Laboratory for Cancer Research, Leidos Biomedical Research, Inc., Frederick, MD 21701 USA; 2 Diller Family Comprehensive Cancer Center, University of California San Francisco, San Francisco, CA 94158 USA

**Keywords:** Molecular biophysics, X-ray crystallography

## Abstract

Although post-translational modification of the C-terminus of RAS has been studied extensively, little is known about N-terminal processing. Mass spectrometric characterization of KRAS expressed in mammalian cells showed cleavage of the initiator methionine (iMet) and N-acetylation of the nascent N-terminus. Interestingly, structural studies on GDP- and GMPPNP-bound KRAS lacking the iMet and N-acetylation resulted in Mg^2+^-free structures of KRAS with flexible N-termini. In the Mg^2+^-free KRAS-GDP structure, the flexible N-terminus causes conformational changes in the interswitch region resulting in a fully open conformation of switch I. In the Mg^2+^-free KRAS-GMPPNP structure, the flexible N-terminus causes conformational changes around residue A59 resulting in the loss of Mg^2+^ and switch I in the inactive state 1 conformation. Structural studies on N-acetylated KRAS-GDP lacking the iMet revealed the presence of Mg^2+^ and a conformation of switch regions also observed in the structure of GDP-bound unprocessed KRAS with the iMet. In the absence of the iMet, the N-acetyl group interacts with the central beta-sheet and stabilizes the N-terminus and the switch regions. These results suggest there is crosstalk between the N-terminus and the Mg^2+^ binding site, and that N-acetylation plays an important role by stabilizing the N-terminus of RAS upon excision of the iMet.

## Introduction

Mutations in RAS proteins are responsible for ~25% of all human cancers. Among the RAS proteins - HRAS, NRAS, and the alternatively spliced KRAS4a and KRAS4b (referred from hereon as KRAS), mutations in KRAS are responsible for 86% of RAS-driven cancers including pancreatic, colorectal, and lung cancers^1,2^. RAS proteins are binary molecular switches that cycle between active guanosine triphosphate (GTP)-bound and inactive guanosine diphosphate (GDP)-bound states. Interconversion between these two states is intrinsically slow. The conversion from the inactive GDP-bound state to the active GTP-bound state is stimulated by guanine nucleotide exchange factors (GEFs). The conversion back to the inactive form is mediated by GTPase-activating proteins (GAPs). In the active state, RAS proteins interact with a variety of effector proteins, such as RAF kinase, PI3K, and RalGDS, leading to activation of downstream signaling pathways. Oncogenic RAS mutations are predominantly found at amino acid positions G12, G13, and Q61.

RAS proteins are members of a superfamily of RAS-related small GTPases that are divided into five major subfamilies based on their sequence and functional similarities: RAS, RHO, RAB, ARF, and RAN^3–5^. All members of the five subfamilies have a common core structure, which consists of a central β-sheet with six strands that is flanked by five α-helices on both sides. The ARF and RAN subfamilies have an additional seventh β-strand in the GDP-bound form that extends the central β-sheet^3^. The role of magnesium as an essential cofactor for small GTPases is well established as it is required for both high-affinity nucleotide binding and GTP hydrolysis^6^. In structures of RAS bound to Mg^2+^ and nonhydrolyzable GTP analogs GMPPNP (GppNHp) or GMPPCP, Mg^2+^ forms a bidentate complex with the β- and γ-phosphates and is required for high-affinity binding of the nucleotide with the protein^7^. Residues S17, T35, and two water molecules are the other coordination partners of the Mg^2+^. In the structures of RAS bound to Mg^2+^ and GDP, two water molecules take the place of γ-phosphate and T35. In the absence of Mg^2+^, intrinsic GTP hydrolysis is undetectable^6^. Structural work on RAS proteins has been limited to Mg^2+^ and GDP/GTP-bound forms; therefore, little is known about RAS structures in the Mg^2+^-free state.

^31^P NMR experiments have shown that, in solution, GTP-bound HRAS exists in an equilibrium between two conformations, “inactive” state 1 and “active” state 2^8–11^. Crystal structures of GTP analog-bound HRAS in complex with various effectors have revealed the state 2 conformation in which the switch I and switch II regions are fixed by interactions of T35 and G60, respectively, with the γ-phosphate of GTP^12^. Conversely, switch I and II regions in state 1 conformation are dynamically mobile on the picosecond to nanosecond timescale^8^. Structural information about the state 1 conformation has been obtained from crystal structures of HRAS mutants T35S, T35A, A59G, and G60A, and MRAS, a member of the RAS subfamily^11,13–16^. In these structures showing the state 1 conformation, T35 (or mutated residue) in HRAS and its equivalent T45 in MRAS are not capable of interacting with the γ-phosphate of GTP, resulting in marked deviation of switch I from the nucleotide-binding pocket. Unlike the state 2 conformation, RAS-GTP in the state 1 conformation forms surface pockets, which may be potentially targeted using small-molecule inhibitors^17^.

In cells, RAS is synthesized as a precursor protein that requires post-translational processing for membrane association and to become biologically active^1,2,18^. The modifications at the C-terminus of RAS isoforms that include farnesylation, palmitoylation, carboxymethylation, and the proteolytic removal of the final three C-terminal residues have been studied extensively^1,2^. There is very little known about the modifications at the N-terminus of RAS and any potential effects these may have on RAS structure and biological activity. The cleavage of the N-terminal methionine residue and N-terminal acetylation are the most common post-translational modifications in eukaryotes^19,20^. Approximately two-thirds of the proteins in any proteome undergo N-terminal methionine excision by the action of specific methionine aminopeptidases, which are present in all organisms from bacteria to eukaryotes^21^. The second residue exposed by the action of the methionine aminopeptidases is often further modified (N-acetylation) by N-acetyltransferase (NAT). N-acetylation is very common in eukaryotes but rarely occurs in prokaryotes^22^. The functional importance of this modification has been suggested that it neutralizes positive charge associated with the free-amino group at the N-terminus, and thereby efficiently blocks its further ionization and modifications^23^. Previous studies and results presented here suggest that cellular KRAS has also been modified such that after initiator methionine (iMet) excision, the N-terminus is acetylated at the second amino acid, threonine^24,25^.

The structural work described here on KRAS lacking the iMet and N-acetylation (not possible in bacterial expression system) in complex with GDP and GMPPNP fortuitously provided Mg^2+^-free structures of RAS proteins. In these structures, lack of the iMet results in flexible N-termini which cause conformational changes that propagate to the switch and interswitch regions, dislodging Mg^2+^ from the nucleotide binding pocket. The structure of N-acetylated KRAS-GDP lacking the iMet (expressed in insect cell expression system) resembles the structure of unprocessed KRAS-GDP containing the iMet. It contains Mg^2+^ and relatively stable switch regions due to stabilization of the N-terminus by the N-acetyl group in the absence of the iMet. The conformational changes observed near the Mg^2+^ binding site in KRAS with different N-termini suggest crosstalk between these two sites.

## Results

### N-terminal methionine excision and N-acetylation of KRAS

**Figure 1:**
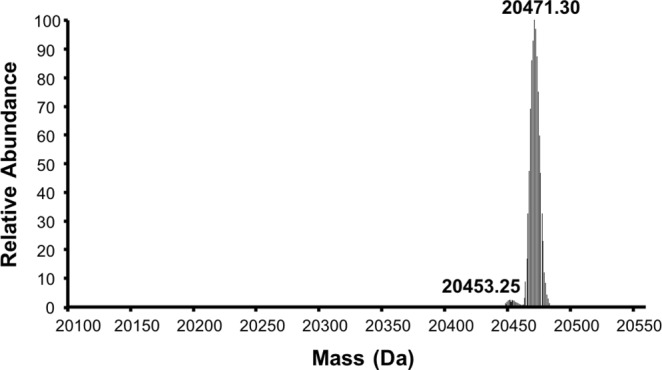
Mass spectrometric  characterization of KRAS4b expressed in HEK293 cells. The electrospray ionization-mass spectrometry (ESI-MS) spectrum of the purified human KRAS4b (1–171 with a C-terminal His8 tag) expressed in HEK293 cells and purified using immobilized metal affinity chromatography. The dominant species at 20,471 Da corresponds to the expected mass of the protein lacking initiator methionine and the presence of N-acetylation at the nascent N-terminus.

Based on the N-terminal modification observed in the vast majority of eukaryotic proteins, KRAS is likely to undergo excision of the iMet followed by N-acetylation of the nascent N-terminus starting with the second amino acid, threonine. Such results were previously reported in studies using a pancreatic cancer cell line PSN-1^24^, and in a colorectal cancer line DLD-1^25^. To confirm these results using a non-transformed cell line, we generated a mammalian expression vector with human KRAS4b (residues 1–171 followed by a His8 tag for purification) and transfected it into HEK293 cells. After 72 hours, cell pellets were harvested, and the proteins was purified. The intact molecular mass of the purified protein was measured by electrospray ionization mass spectrometry (ESI-MS). The expected molecular masses of the KRAS (1–171)-His8 and its modifications are 20,561 Da (intact protein); 20,429 Da (protein lacking iMet); 20,471 Da (protein lacking the iMet with N-terminal acetylation). ESI-MS analysis of the protein confirmed that the N-terminal modifications in KRAS involves cleavage of the iMet and N-acetylation of the nascent N-terminus (Fig. 1 and Supplementary Fig. 1a,b). Despite expressing KRAS at levels that far exceed the normal levels^26^, the protein processing machinery in HEK293 cells were able to modify essentially all of the recombinant KRAS at the N-terminus. Our results support the previous observation that the mammalian cellular machinery is able to fully process the N-terminus of KRAS by excision of the iMet followed by N-acetylation of the nascent N-terminus^24,25^.

### The crystal  structure of GDP-bound KRAS (2–169) has a fully open switch I conformation and lacks Mg^2+^

Based on the N-terminal processing observed in KRAS, a KRAS G-domain construct was made beginning with the second amino acid (threonine) to either residue 166, or 169. Recombinant proteins made with these constructs using bacterial (*E. coli*) expression system lacked N-acetylation as prokaryotes do not contain N-α-terminal acetyltransferase complexes that are required for this modification. Recombinant proteins were used for crystallization of KRAS in the inactive (GDP-bound) and active states (bound to a nonhydrolyzable analog of GTP, GMPPNP) in the presence of Mg^2+^. We first solved the structures of GDP-bound KRAS using the two KRAS constructs 2–166 and 2–169. Both KRAS constructs crystallized in the same crystal form. Since we obtained higher resolution data (1.5 Å) using KRAS (2–169) and the electron density was observed up to residue 169, we selected this structure for subsequent analysis (Supplementary Tables 1 and 2).

**Figure 2:**
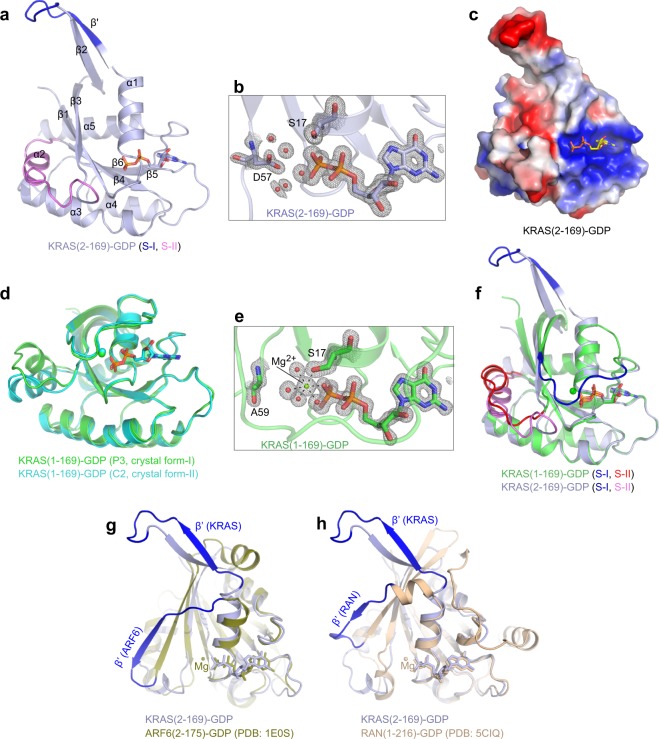
Crystal structure of Mg^2+^-free KRAS (2–169)-GDP and structural comparison with Mg^2+^-bound KRAS (1–169)-GDP and members of the RAS superfamily. (**a**) Overall structure of Mg^2+^-free KRAS (2–169) bound to GDP shown in ribbon representation. GDP is shown in stick representation and switch I and II regions are highlighted in blue and violet, respectively. Secondary structural elements (α1–α5 and β1–β6) including the additional β-strand (β’) are labeled. (**b**) Enlarged view of the nucleotide binding pocket in KRAS (2–169)-GDP structure showing electron density map (*2Fo-Fc* contoured at 1.5σ) for GDP, S17, D57, and water molecules. (**c**) Electrostatic surface presentation of Mg^2+^-free KRAS (2–169)-GDP. Acidic, basic and neutral regions are shown in red, blue, and white, respectively. (**d**) Structural superposition of Mg^2+^-bound KRAS (1–169)-GDP structures obtained in two different crystal forms showing similar conformation for the switch I region. (**e**) Enlarged view of the nucleotide binding pocket in KRAS (1–169)-GDP structure showing electron density map (*2Fo-Fc* contoured at 1.5σ) for GDP, S17, A59, Mg^2+^, and water molecules. (**f**) Structural comparison of Mg^2+^-free and Mg^2+^-bound structures of KRAS-GDP showing large conformational change in the switch I region in the Mg^2+^-free structure of KRAS-GDP. (**g**,**h**) Structural superposition of the Mg^2+^-free KRAS structure with Mg^2+^/GDP-bound (**g**) ARF6 (PDB: 1E0S) and (h) RAN (PDB: 5CIQ) showing the presence of an additional β-strand (β’) in switch I (highlighted in blue).

The overall structure of the GDP-bound KRAS (2–169) is shown in Fig. 2a. Surprisingly, no electron density was observed for the Mg^2+^ (Fig. 2b), and switch I is in a fully open conformation with residues 26–30 forming an additional β-strand denoted as β’. The β’-strand forms an antiparallel β-sheet with the β2 strand which is no longer a part of the central β-sheet. To date, all structures of RAS isoforms in complex with GDP have Mg^2+^ coordinated octahedrally with the β-phosphate of GDP, residue S17, and water molecules. In the absence of Mg^2+^, the S17 side chain atom points away from the β-phosphate of GDP. We refer to this structure as Mg^2+^-free KRAS-GDP. An electrostatic surface representation of Mg^2+^-free KRAS-GDP reveals a new shallow pocket between the switch I region and the nucleotide binding pocket that may be amenable to structure-based drug discovery approaches (Fig. 2c). A comparison of the structure of GDP-bound KRAS (2–166) with GDP-bound KRAS (2–169) shows an identical conformation for KRAS, including a fully open switch I conformation and no electron density corresponding to a Mg^2+^. Analysis based on structures of RAS in the Protein Data Bank suggest that the absence of the iMet in the KRAS (2–169) construct is likely the reason for the fully open conformation of switch I and the lack of Mg^2+^.

To understand the role of the iMet, we made a new KRAS expression construct (1–169) that included the iMet and crystallized it in complex with GDP. Crystal structures of GDP-bound KRAS (1–169) in two different crystal forms were solved, both diffracting to a resolution of 1.45 Å (Supplementary Tables 1 and 2). Structural superposition of the two crystal forms of GDP-bound KRAS (1–169) shows a similar conformation for the switch I (partially closed and interacting with the nucleotide binding pocket) and the presence of Mg^2+^ as seen in the KRAS-GDP structures (PDB IDs 4LPK and 4OBE) solved previously^25,26^ (Fig. 2d,e). From hereon we refer to these two structures as the Mg^2+^-bound KRAS-GDP structure. We observed a minor difference in the switch II conformation in these two crystal forms, suggesting stabilization of the flexible switch II region by different crystal contacts (Fig. 2d). A structural superposition of Mg^2+^-free KRAS-GDP with Mg^2+^-bound KRAS GDP structures shows large conformational changes not only in the switch I but also in the interswitch region (Fig. 2f).

Structural analysis suggests that the lack of either iMet or N-terminal acetylation (not present in the recombinant protein used in this experiment) is likely to be responsible for the fully open switch I conformation and the lack of Mg^2+^. Surprisingly, an oncogenic A146T mutant of KRAS (1–169) bound to GDP crystallizes in the same crystal form as Mg^2+^-free KRAS (2–169)-GDP and has an identical fully open switch I conformation with no Mg^2+^ in the nucleotide binding pocket (Kent Rossman, personal communication). A recent study also showed similar structural changes in the A146T mutant of KRAS bound to GDP^27^. These observations suggest this conformation is captured inside the crystal due to rearranged intramolecular interactions within the G-domain of KRAS that can be triggered either by an oncogenic mutation such as A146T or modification at the N-terminus (lack of either the iMet or N-acetylation). Inside the crystal, the fully open switch I conformation has minimal interaction with neighboring molecules and is primarily stabilized by two hydrogen bonds formed by residues Y32 and Y40 present in the loop between the new β-strand and β2 with the residues Q61 and Y64, respectively, from the symmetry-related molecule (Supplementary Fig. 2). Previously, GDP/Mg^2+^-bound structures of ARF6^28^ and RAN^3,29^, members of the RAS superfamily, have been observed with an additional β-strand in the switch I region. This suggests the formation of the additional β-strand is not due to crystal packing interactions but likely to be an inherent property of switch I region, in at least some of the RAS superfamily members (Fig. 2g,h). In ARF6/RAN, the additional β-strand is part of the central β-sheet, whereas in KRAS it forms an antiparallel β-sheet with the β2-strand that is no longer a part of the central β-sheet.

### Structural and biochemical analysis of Mg^2+^-free KRAS (2–169)-GDP and Mg^2+^-bound KRAS (1–169)-GDP

**Figure 3:**
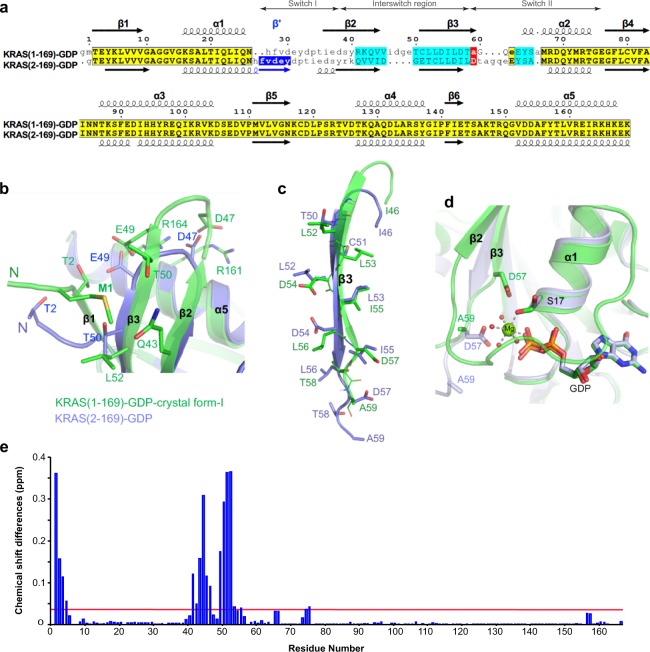
Structural comparison between Mg^2+^-free and Mg^2+^-bound KRAS-GDP structures showing conformational changes in the switch and interswitch regions. (**a**) Structure-based sequence alignment between Mg^2+^-free KRAS (2–169)-GDP and Mg^2+^-bound KRAS (1–169)-GDP showing the presence of an additional β strand (β’, highlighted in blue) in the switch I region and the two-residue shift (highlighted in cyan) in the β2 and β3 strands present in the interswitch region. Secondary structural elements, helices and strands, are shown as spirals and arrows, respectively. Residues highlighted in yellow align well in Mg^2+^-free and Mg^2+^-bound KRAS structures. (**b**,**c**) Structural superposition of Mg^2+^-free and Mg^2+^-bound KRAS-GDP showing the displaced N-terminus in the Mg^2+^-free KRAS-GDP structure resulting in (**b**) smaller β2 and β3 strands and (**c**) a two amino acid shift in the β3 strand. (**d**) Structural superposition of Mg^2+^-free and Mg^2+^-bound KRAS-GDP showing the presence of D57 in place of A59 in the Mg^2+^-free KRAS-GDP structure resulting in stearic clash with water-coordinated Mg^2+^. Water molecules are shown as small red spheres. (**e**) Comparison of HSQC spectra obtained for GDP-bound KRAS (2–169) and KRAS (1–169). Histogram of normalized ^1^H-^15^N chemical shift changes vs residue number obtained from the HSQC spectra of ^15^N KRAS (1–169) vs ^15^N KRAS (2–169). Red horizontal line represents average chemical shift differences measured.

A structure-based sequence alignment of Mg^2+^-free KRAS (2–169)-GDP and Mg^2+^-bound KRAS (1–169)-GDP shows that all residues except those present in the switch and interswitch regions align with each other (Fig. 3a). The non-aligned region includes switch I, strands β2, β3 and the intervening loop, as well as the switch II region. The structural superposition shows that in the Mg^2+^-bound KRAS (1–169)-GDP structure, the iMet interacts with residues L52, T50 and Q43 (present on β2 and β3 strands) via hydrophobic and van der Waals interaction (Fig. 3b). The lack of the iMet in Mg^2+^-free KRAS (2–169)-GDP structure results in residues T2 and E3 forming a loop structure (instead of being part of the β1 strand) that points away from the central β-sheet (Fig. 3b). A smaller β1 strand results in smaller β2 and β3 strands within the central β-sheet and as a result, residues within the turn between β2-β3 strands and the β3 strand (interswitch region) undergo a register shift by two amino acids (Fig. 3c). This has been seen previously in the case of ARF6 (see “Discussion”). Despite the two amino acid shift, the backbone atoms of residues present on the β3 strand maintain hydrogen-bonding interactions with β1 and maintain the pattern of side-chain hydrophobicity at most positions. It is likely that the two amino acid shift results in the loss of existing intramolecular interactions between side chain atoms of the β2 and β3 strands. Once the β2 strand is detached from the central β-sheet, it is likely to make switch I more flexible resulting in a fully open conformation. In the Mg^2+^-free KRAS-GDP structure, the fully open conformation of switch I with an additional β-strand forming an antiparallel β-sheet may represent a low energy conformation that is captured inside the crystal. A register shift of two amino acids in the β3 strand results in replacement of residue A59 with D57. A structural superposition shown in Fig. 3d shows that the side chain atoms of D57 sterically clash with the water molecules coordinated to Mg^2+^ resulting in displacement of Mg^2+^ from the nucleotide binding pocket.

To examine the secondary structural content of KRAS in the presence and absence of the iMet, we examined the GDP-bound KRAS (1–169) and KRAS (2–169) using circular dichroism (Supplementary Fig. 3a) and thermal melt analysis. Results obtained from these experiments showed similar secondary structural content as well as melting temperature (Tm) of 56.5 ± 0.5 °C for these two proteins, suggesting that they have similar secondary and tertiary structures in solution. We examined the two KRAS constructs for their intrinsic GTP hydrolysis activity as well as for interaction with the RAS-binding domain (RBD) of RAF1. ^32^P-based intrinsic GTP hydrolysis experiments showed a similar rate for both KRAS constructs (Supplementary Fig. 3b). Isothermal titration calorimetry experiments showed that both KRAS constructs bind to RAF1-RBD with similar affinity, suggesting that biochemical and biophysical properties of these two KRAS constructs are very similar (Supplementary Fig. 4a,b).

To determine whether the structural changes observed in the crystal structure of Mg^2+^-free KRAS-GDP are observed in solution, 2D ^1^H-^15^N Heteronuclear Single Quantum Coherence (HSQC)-NMR experiments were carried out using ^15^N labeled GDP-bound KRAS (1–169) and KRAS (2–169). Comparison of the NMR peaks obtained for these proteins shows a large chemical shift perturbation for residues at the N-terminus, and for the β2-β3 strands present in the interswitch region (Fig. 3e and Supplementary Fig. 5). No chemical shift perturbations were observed for the residues present in the switch regions. NMR chemical shift changes are very sensitive to their chemical environment, however residues that are solvent exposed in different conformational states experience the same environment^30^. Therefore, the absence of the chemical shift differences in the switch regions are likely due to these areas being solvent exposed in both of these proteins. Previous NMR spectroscopic study has shown that the switch regions of HRAS-GDP have large conformational flexibility in solution and show intrinsic mobility on the nanosecond timescale^31^.

Considering Mg^2+^ is required for high-affinity nucleotide binding, we measured the rate of nucleotide exchange in KRAS with and without the iMet in the presence and absence of SOS (Son of Sevenless). In the presence of Mg^2+^ in the reaction mixture, KRAS proteins (1–169 and 2–169) showed similar rates for intrinsic and SOS-mediated nucleotide exchange (Supplementary Fig. 6). However, in the absence of Mg^2+^ in the reaction mixture, both KRAS proteins showed intrinsic exchange rates higher than the SOS-mediated exchange rate obtained in the presence of Mg^2+^. When SOS was added to this Mg^2+^-free reaction mixture, the reaction occurred so rapidly that the majority of the nucleotide was displaced before the first measurement was made. Although KRAS proteins with and without the iMet showed similar rates of intrinsic and SOS-mediated nucleotide exchange, these results suggest that the loss of Mg^2+^ in KRAS increases the rate of intrinsic and SOS-mediated nucleotide exchange significantly, possibly due to decreased affinity of nucleotide and greater structural flexibility of the switch regions.

### The crystal  structure of GMPPNP-bound KRAS (2–166) reveals no Mg^2+^ and switch I in the state 1 conformation

**Figure 4:**
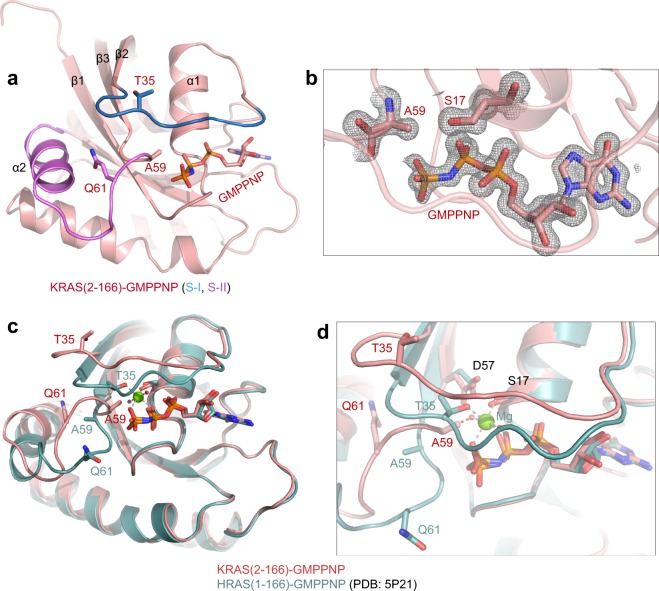
Structure of Mg^2+^-free KRAS in the GMPPNP-bound state and structural comparison with Mg^2+^-bound HRAS-GMPPNP structures. (**a**) Crystal structure of Mg^2+^-free KRAS (2–166)-GMPPNP. GMPPNP and residues T35, A59 and Q61 are shown in stick representation and switch I and II regions are highlighted in navy blue and violet, respectively. (**b**) Enlarged view of the nucleotide binding pocket in KRAS (2–166)-GMPPNP structure showing electron density map (*2Fo-Fc* contoured at 1.2 σ) for GMPPNP, S17 and A59. (**c**) Structural superposition of Mg^2+^-free KRAS-GMPPNP with Mg^2+^-bound HRAS (1–166)-GMPPNP (PDB: 5P21). (**d**) Enlarged view of switch and interswitch regions showing conformational changes in the key residues T35, A59, and Q61. In Mg^2+^-free KRAS structure, side-chain atoms of residue A59 clash with Mg^2+^-coordinated water molecules (shown as red spheres).

To determine the structural impact of lack of iMet on active KRAS, we attempted to solve the structure of KRAS bound to GMPPNP and Mg^2+^. Among the two KRAS constructs made, we obtained crystals using KRAS (2–166) that diffracted to a resolution of 1.5 Å. The overall structure of KRAS (2–166)-GMPPNP is shown in Fig. 4a. Like the Mg^2+^-free KRAS-GDP structure, no electron density corresponding to Mg^2+^ was observed in this structure (Fig. 4b). From hereon, we refer to this structure as Mg^2+^-free KRAS-GMPPNP. Unlike the Mg^2+^-free KRAS-GDP structure, the switch I region in Mg^2+^-free KRAS-GMPPNP structure is close to the nucleotide binding pocket. Also, in this structure, we did not see a register shift of amino acids in the β2-β3 strands present in the interswitch region. This is likely due to interactions between the gamma-phosphate and residues present in the β3 strand. The lack of interaction between the gamma-phosphate and T35 and the absence of Mg^2+^ results in the inactive state 1 conformation of the switch I region.

### Structural changes responsible for the lack of Mg^2+^ in the Mg^2+^-free KRAS-GMPPNP structures

Structural comparison of Mg^2+^-free KRAS (2–166)-GMPPNP with previously solved Mg^2+^-bound HRAS (1–166)-GMPPNP (the switch I region in active state 2 conformation that is compatible for effector binding) shows that partial closure of switch I and a structural rearrangement at the beginning of the switch II region causes the side chain atoms of Q61 to flip away from the nucleotide binding pocket (Fig. 4c,d). This conformational change in Q61 affects the conformation of the preceding residues G60 and A59 as well. In the Mg^2+^-free KRAS-GMPPNP structure, side chain atoms of A59 point towards the Mg^2+^ and sterically clash with water molecules coordinated with Mg^2+^ (Fig. 4d). This clash results in the loss of Mg^2+^ from this structure.

**Figure 5:**
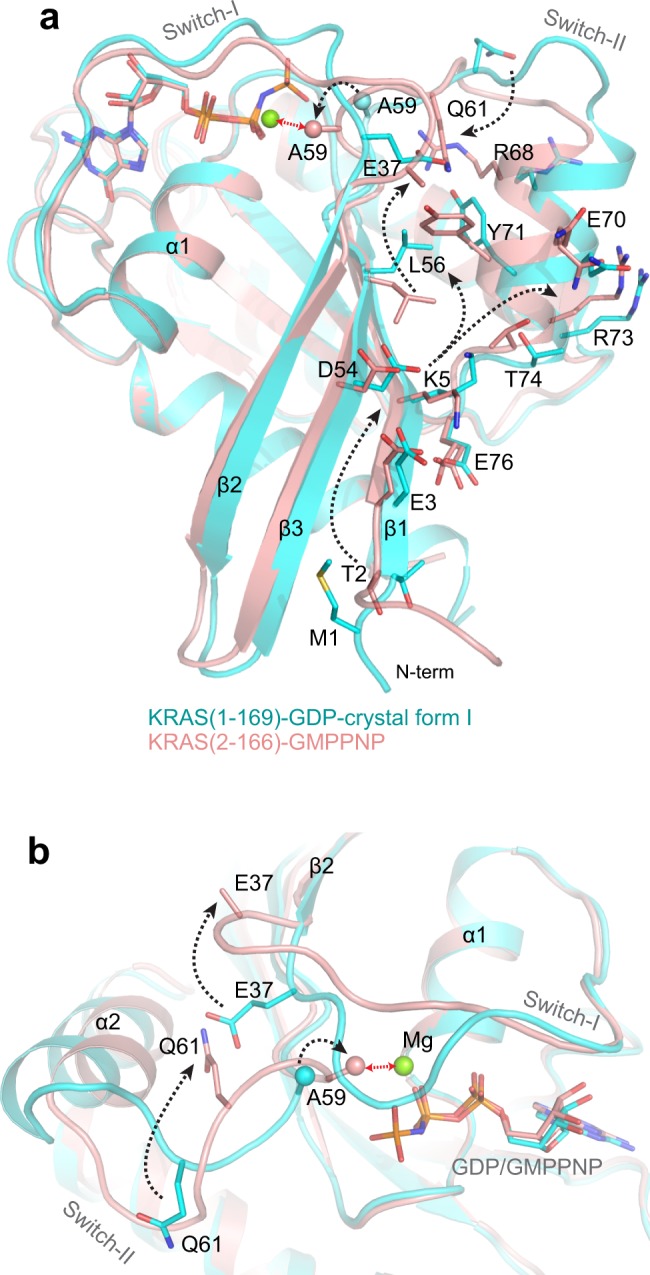
Structural analysis showing conformational changes responsible for lack of Mg^2+^ ion in the KRAS-GMPPNP structure. (**a**) Structural superposition of Mg^2+^-free KRAS-GMPPNP with Mg^2+^-bound KRAS-GDP, showing conformational changes in the side-chain atoms of residues present in β3 strand and switch regions. For clarity, only one crystal form of Mg^2+^-bound KRAS-GDP (cyan) is shown in this panel. Conformational changes originating from the N-terminus propagate to the end of switch I region and beginning of switch II region, resulting in large conformational changes in residues E37 and Q61. (**b**) Enlarged view of the nucleotide binding pocket and switch regions highlighting conformational changes in the superposed structures shown in panel a. The 180° flip in the side chain conformation of Q61 results in rearrangement of residues G60 and A59, causing the side chain atoms of A59 to point towards the octahedrally coordinated Mg^2+^ ion, resulting in a stearic clash (shown as red arrow) with water molecules coordinated with the Mg^2+^. Conformational changes originating from the N-terminal end to residue A59 are shown using dotted arrows. The C-beta atom of residue A59 is shown as a sphere.

Structural comparison of Mg^2+^-free KRAS-GMPPNP with Mg^2+^-bound KRAS-GDP shows similar conformational changes at the N-terminus. (Supplementary Fig. 7). In the absence of iMet in the Mg^2+^-free KRAS-GMPPNP structure, residues present at the N-terminus are displaced from the central β-sheet, as observed in the Mg^2+^-free KRAS-GDP structure (Fig. 5a). Conformational changes observed at the N-terminus propagate to residue E37 via residues present in β1, β3, and switch II. These conformational changes result in a large movement of the E37 side chain, which now points towards the solvent instead of pointing towards the switch II region (Fig. 5b). The space vacated by E37 is taken by Q61 flipping its side chain by almost 180° and pointing away from the nucleotide binding pocket. The side chain flip of Q61 results in conformational changes at the two preceding residues A59 and G60. Side chain atoms of A59 now point towards the Mg^2+^ and sterically clash with water molecules coordinated with the Mg^2+^, thus preventing Mg^2+^ binding to the nucleotide binding pocket (Fig. 5b).

### The crystal structure of N-acetylated KRAS-GDP lacking the iMet contains Mg^2+^ and resembles Mg^2+^-bound KRAS (1–169)-GDP structure

**Figure 6:**
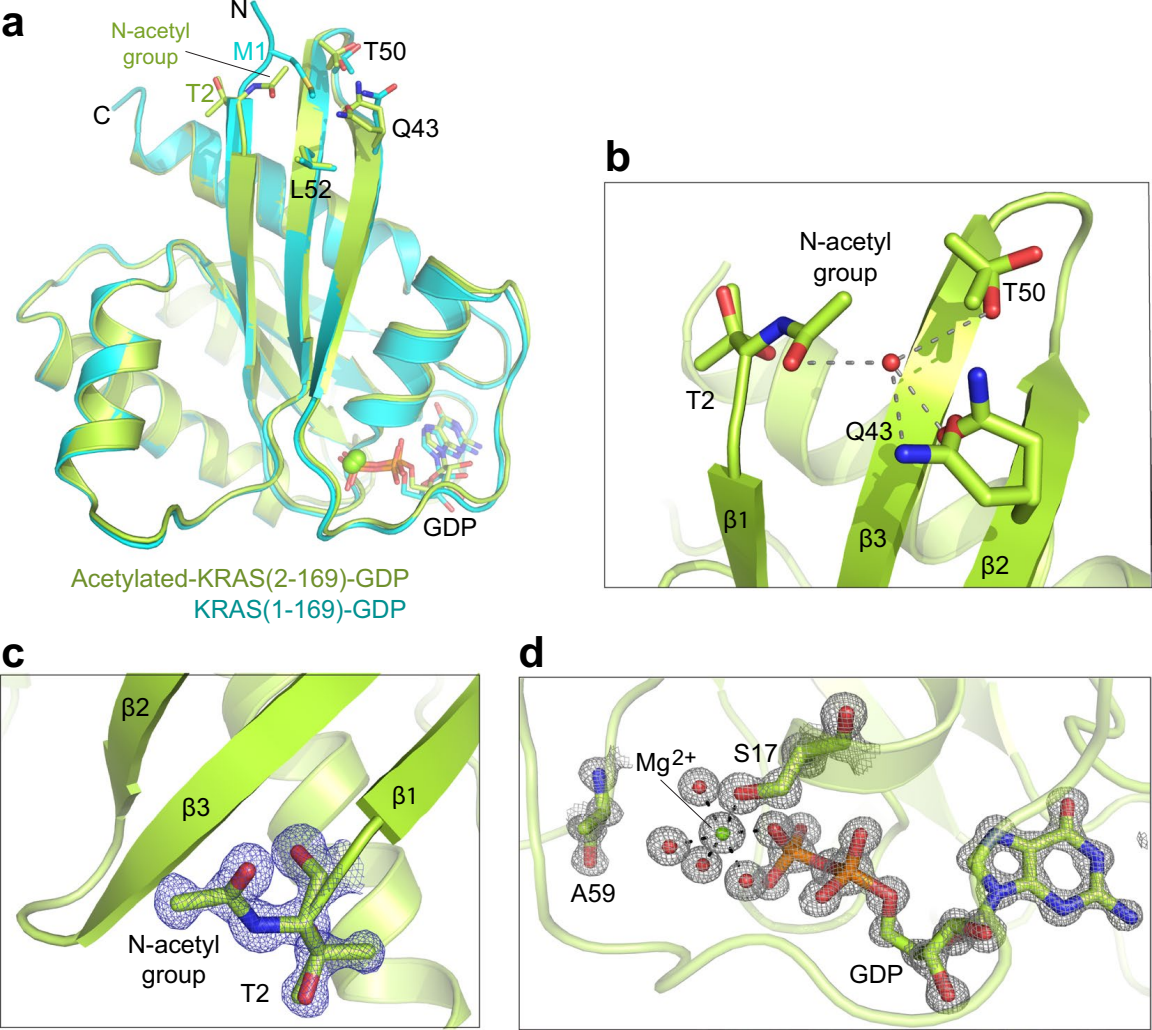
Crystal structure of GDP-bound N-acetylated KRAS lacking the iMet showed presence of Mg^2+^ and interaction between N-acetyl group and neighboring residues present on β2 and β3 strands. (**a**) Structural superposition of N-acetylated KRAS (2–169)-GDP with KRAS (1–169)-GDP showing similar tertiary structure and presence of Mg^2+^ in the nucleotide binding pocket. Initiator methionine and N-acetyl group points towards β2 and β3 strands and interact with residues Q43, T50 and L52. (**b**) Enlarged view of the interaction formed by N-acetyl group with Q43 and T50 mediated by bridging water molecule (red sphere) in the N-acetylated KRAS (2–169)-GDP structure. (**c**,**d**) Enlarged view of the N-terminus and nucleotide binding pocket in N-acetylated KRAS (2–169)-GDP structure showing electron density map (*2Fo-Fc* contoured at 2 σ) for (**c**) N-acetylated T2 and (**d**) GDP, S17, A59, Mg^2+^ and water molecules (red spheres).

Given our observations that KRAS lacking the iMet resulted in Mg^2+^-free structures, we decided to examine the structural role of N-acetylation on the tertiary structure of KRAS. Toward this end, we expressed and purified N-terminal processed KRAS (lacking the iMet with N-acetylation at the nascent N-terminus) using an insect cell expression system. Mass-spectrometric analysis confirmed the cleavage of the iMet and N-acetylation of T2. We attempted crystallization of N-acetylated KRAS (2–169) in both GDP and GMPPNP bound states; however, we could only obtain crystals in the GDP-bound state which diffracted to a resolution of 1.00 Ang (Supplementary Tables 1 and 2**)**. The structure of N-acetylated KRAS-GDP contains Mg^2+^ and resembles the Mg^2+^-bound KRAS (1–169)-GDP structure (Fig. 6a,b). In the structure we see clear electron density for the N-acetylated threonine and the Mg^2+^ present in the nucleotide binding pocket (Fig. 6c,d). The acetyl group interacts with residues Q43 and T50, in the β2 and β3 strands, via water mediated hydrogen bonds, which stabilizes the N-terminus in a similar fashion as the iMet in case of Mg^2+^-bound KRAS (1–169)-GDP structure (Fig. 6b). These results suggest that the presence of either N-acetylation in N-terminally processed KRAS or iMet in unprocessed KRAS is required for stabilizing N-terminus and the central β-sheet.

### Structures of Mg^2+^-free KRAS resemble nucleotide- and Mg^2+^-free HRAS bound to the catalytic pocket of SOS

**Figure 7:**
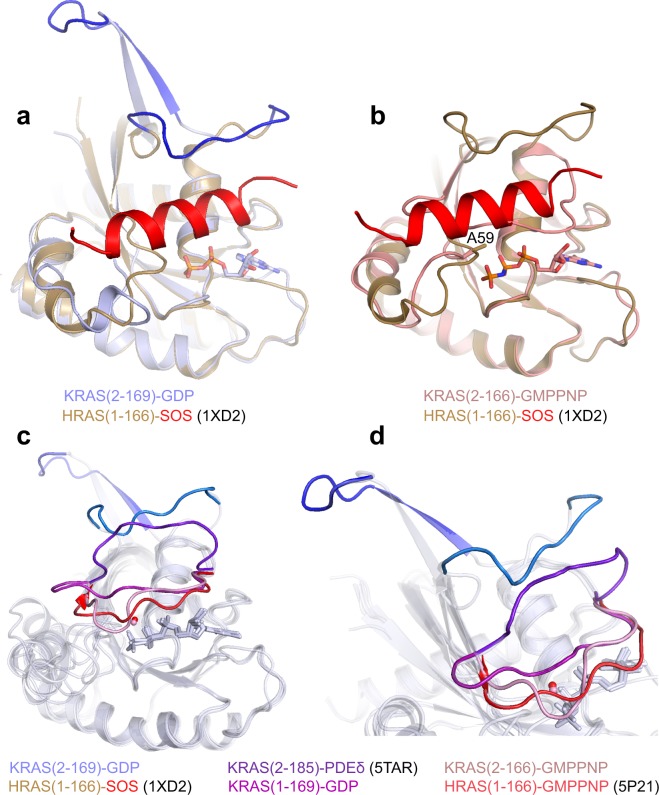
Conformation of switch I seen in Mg^2+^-free KRAS structures provides insights into GEF-mediated nucleotide exchange process and inherent flexibility of the switch I and interswitch region. (**a**) Structural superposition of Mg^2+^-free KRAS-GDP with nucleotide- and Mg^2+^-free HRAS-complexed with SOS (red helix) in the catalytic pocket showing large conformational changes in the switch I region. (**b**) Structural superposition of Mg^2+^-free KRAS-GMPPNP with nucleotide- and Mg^2+^-free HRAS-complexed with SOS showing similar side-chain orientations for residue A59 resulting in Mg^2+^-free RAS structures. (**c**) Structural superposition of three Mg^2+^-free KRAS structures (solved in different crystal forms) KRAS (2–169)-GDP, KRAS(2–166)-GMPPNP, and prenylated KRAS (2–185)-GDP bound to PDEδ with nucleotide- and Mg^2+^-free HRAS(1-166) complexed with SOS, KRAS(1-169) bound to GDP/Mg^2+^, and HRAS(1-166) bound to GMPPNP/Mg^2+^ showing large inherent flexibility in the switch I and interswitch region. (**d**) Enlarged view of the switch I region showing five snapshots between open and closed conformations. Structural details and color scheme are written below panels (c,d).

The absence of Mg^2+^ and the fully open conformation of switch I observed in the Mg^2+^-free KRAS-GDP structure resembles the open conformation of switch I seen in the structure of nucleotide- and Mg^2+^-free HRAS bound to the catalytic pocket of SOS (Fig. 7a). In the nucleotide- and Mg^2+^-free HRAS^32^, the space between switch I and the nucleotide binding pocket is occupied by a SOS helix that plays a key role in dissociating Mg^2+^ and GDP from RAS. Our Mg^2+^-free KRAS-GDP structure corroborates the previous observation that SOS first displaces Mg^2+^ from the nucleotide-binding pocket, resulting in a flexible switch I region, and then the SOS helix binds between switch I and the nucleotide binding pocket by displacing the bound GDP^33^.

A structural comparison of nucleotide- and Mg^2+^-free HRAS bound to the SOS catalytic pocket with the Mg^2+^-free KRAS-GMPPNP shows similar side chain conformations for residue A59 in both RAS structures (Fig. 7b). This suggests that conformational changes in the switch II region of HRAS occurring upon binding to SOS result in the side chain atoms of A59 pointing towards Mg^2+^ and displacing it from its position. Thus, the structure of Mg^2+^-free KRAS-GMPPNP provides a probable intermediate snapshot of the SOS-mediated nucleotide exchange process where it likely displaces the Mg^2+^ using residue A59 before displacing GDP.

### Structures of Mg^2+^-free KRAS provide insights into the inherent flexibility of the switch I region

The structural superposition of Mg^2+^/GDP-bound KRAS(1-169) and Mg^2+^-free KRAS(2-166/169) structures bound to GDP and GMPPNP with previously reported Mg^2+^-free farnesylated and methylated KRAS(2–185)-GDP in complex with PDEδ^34^, nucleotide- and Mg^2+^-free HRAS bound to SOS^32^, and Mg^2+^/GMPPNP-bound HRAS^7^ show six  different snapshots of switch I (Fig. 7c,d). These conformations of switch I range from fully open, as in the Mg^2+^-free KRAS-GDP structure, to the fully closed effector-binding conformation (state 2 and active), as seen previously in the Mg^2+^-bound HRAS-GMPPNP structure (PDB: 5P21). The switch I conformations seen in the other three structures provide snapshots between these two conformations indicating the large inherent flexibility present in switch I.

## Discussion

In eukaryotes, the excision of the iMet and N-terminal acetylation are two of the most common protein post-translational modifications^19,20^. These two modifications contribute to proteomic diversity and complexity and have been suggested to play an important role in protein regulation and cellular signaling^35^. Previously, mass spectrometric characterization of KRAS expressed in pancreatic and colorectal cancer cells have shown that KRAS, like most eukaryotic proteins, undergoes N-terminal methionine excision and N-terminal acetylation^24,25^. Mass spectroscopic characterization of KRAS expressed in mammalian cells presented here further supported this observation. Considering all four RAS isoforms have identical residues at the N-terminus and the first half of the G-domain, it is likely that they all undergo the same N-terminal processing. To examine the effect of N-terminal modification on the tertiary structure of KRAS, we carried out structural studies on KRAS constructs containing iMet (unprocessed N-terminus), lacking the iMet (partially processed N-terminus), and KRAS lacking the iMet and with N-acetylation of the second amino acid (fully processed N-terminus). Crystal structures of KRAS (2–169) lacking the iMet resulted in Mg^2+^-free structures in the GDP- and GMPPNP-bound forms. In the Mg^2+^-free KRAS-GDP structure, a register shift by two residues in the interswitch region results in a fully open conformation of switch I. In this conformation, a new β-strand forms in the switch I region as seen previously in ARF6 and RAN. In the Mg^2+^-free KRAS-GMPPNP structure, a flexible N-terminal region results in conformational changes that propagate to residue A59 that dislodges Mg^2+^ and in turn adopts an inactive state 1 conformation of the switch I region. These Mg^2+^-free KRAS structures, for the first time, suggest the inherent flexibility of the interswitch region, and an increased flexibility of the switch I region in comparison to what  has been predicted from earlier biophysical and structural analysis of RAS proteins^11,15^.

The 1.0 Ang structure of GDP-bound N-acetylated KRAS (2–169) provides atomic details of the interactions formed by N-acetyl group with the central β-sheet. This structure showed the presence of Mg^2+^ in the nucleotide binding pocket as observed in the structures of unprocessed KRAS (1–169). Structural data presented here show that the N-acetyl group and the iMet play a similar role in stabilizing the N-termini and the central β-sheet of their respective proteins, N-terminally processed KRAS (2–169) and unprocessed KRAS (1–169). These results suggest the importance of N-acetylation in KRAS and possibly in other RAS isoforms, where its role is to stabilize the N-terminal end after cleavage of the iMet which in turn prevents any conformational changes around the Mg^2+^ binding site. The lack of either N-acetylation in fully processed KRAS or the iMet in unprocessed KRAS results in conformational changes that propagate to the nucleotide binding pocket, and dislodges Mg^2+^ bound to the nucleotide.

The structural similarity between the GDP-bound structures of an oncogenic A146T mutant of KRAS (1–169) containing iMet and wild-type KRAS (2–169) without the iMet suggests that the structure of Mg^2+^-free KRAS-GDP described here resembles the conformation of KRAS that exists in oncogenic mutants such as A146T^27^. It is important to note here that in the wild type KRAS structure, residue A146, the N-terminus, and the Mg^2+^ binding site are far from each other. Thus, these observations suggest that small perturbations in KRAS such as the lack of N-acetylation or the A146T mutation can cause an allosteric change in the nucleotide binding pocket as well as in the switch and interswitch regions.

Unlike structures solved by NMR, interpretation of crystal structures of proteins with flexible regions captured in a fixed conformation requires careful analysis to determine if the conformation observed in the crystal is of physiological relevance. Validation using other solution-based studies and obtaining structures in multiple crystal forms (with different crystal packing interactions) helps in the correct interpretation of a biological process. Using a partially processed KRAS construct (2–169) lacking the iMet, we obtained four different crystal forms, one of which contains two copies of KRAS in the asymmetric unit. In all of these structures, we saw no electron density corresponding to Mg^2+^. The four crystal forms include the structure of KRAS (2–169) with GDP and GMPPNP described here and our previous work on farnesylated and methylated KRAS (2–185)-GDP in complex with PDEδ^31^. Thus, multiple structures of KRAS obtained in different crystal forms using KRAS constructs lacking the iMet and N-acetylation suggest that the absence of Mg^2+^ in these structures is not due to crystal packing interactions but likely due to allosteric changes caused by modifications at the N-terminus. Analysis of NMR and crystallographic results for KRAS (2–169) and KRAS (1–169) constructs suggests that KRAS lacking the iMet and N-acetylation has  a higher flexibility in the switch and interswitch regions. Considering recombinant KRAS proteins with and without the iMet have similar biochemical properties, it is likely that, in solution, KRAS without the iMet has relatively high flexibility in the switch and interswitch regions resulting in conformational states (with nanosecond mobility) that range from the fully open switch I conformation (Mg^2+^-free state) to the closed switch I conformation (Mg^2+^-bound state).

Biophysical and structural studies on T35 mutants of HRAS have shown that the switch I region has inherent flexibility^11^. In the absence of the iMet as well as N-acetylation, the switch I region of Mg^2+^-free KRAS (2–169)-GDP structure may resemble T35 mutants in solution. However, unlike T35 mutants of HRAS, the lack of Mg^2+^ in the Mg^2+^-free KRAS-GDP structure further enhances the flexibility of the switch I region. To our knowledge, the open conformation of switch I observed in the Mg^2+^-free KRAS-GDP structure is farthest from the nucleotide binding pocket in all the small GTPase structures solved thus far. The open conformation of switch I seen in the Mg^2+^-free KRAS-GDP structure supports previous molecular dynamics studies carried out on Mg^2+^-free RAS, RHO, RAB, ARF, and RAN proteins that suggested Mg^2+^-free small GTPases contain a groove between the switch region and the nucleotide binding site^36^. Previously, the crystal structure of Mg^2+^-free RHOA-GDP revealed the switch I region dislocated from the nucleotide-binding site^37^. This study also suggested that Mg^2+^-free RHOA could be an intermediate of the GEF-mediated GDP/GTP exchange reaction. Structural superposition of Mg^2+^-free KRAS-GDP and Mg^2+^-free RhoA-GDP shows that unlike KRAS, the RHOA switch I does not form any additional β-strands and the extent of its movement from the nucleotide-binding site is much less.

The presence of an additional β-strand in switch I has now been seen in structures of KRAS, ARF6 and RAN, members of three different RAS subfamilies^3,28^. Structural comparison of Mg^2+^-free KRAS and Mg^2+^-bound ARF6 in GDP- and GMPPNP-bound states show similar conformational changes in the GDP-bound state. Like the Mg^2+^-bound ARF6-GDP structure, the structure of Mg^2+^-free KRAS-GDP shows the presence of an additional β-strand the in switch I region and smaller β2-β3 strands resulting in a two amino acid shift in the β3 strand. In the case of ARF6, the amino acid shift in the β2-β3 turn and the β3 strand results in reorientation of the N-terminal helix to its membrane-bound conformation in the GTP bound state. Thus, conformational changes observed in the switch and interswitch regions in ARF6 play an important role in ARF6 interaction with the membrane (Supplementary Fig. 8a,b). It will be interesting to uncover the role of the amino acid shift in the interswitch region of KRAS when it has an A146T mutation.

In the GEF-mediated nucleotide exchange reactions of RAS and ARF, the specific GEFs SOS and ARNO (ARF nucleotide-binding site opener) appear to promote the dissociation of GDP in part through destabilizing the interaction between Mg^2+^ and the GTPase^33,38^. In the Mg^2+^-free KRAS-GDP structure, the absence of Mg^2+^ and the open conformation of switch I show similarities with nucleotide- and Mg^2+^-free HRAS bound to catalytic pocket of SOS. It is possible that the switch I region is in a fully open conformation with an additional β-strand during the initial steps of GEF-mediated nucleotide exchange process. The Mg^2+^-free KRAS structures support the mechanism of SOS-mediated nucleotide exchange in which Mg^2+^ and subsequently the phosphates of GDP are released upon SOS binding. The Mg^2+^-free KRAS structures reveal an important role of the Mg^2+^ in the SOS-mediated nucleotide exchange process. The similar conformations of the A59 residue in the Mg^2+^-free KRAS structures described here and nucleotide- and Mg^2+^-free HRAS bound to SOS suggests that SOS utilizes this mechanism to displace Mg^2+^ and generate an open conformation of switch I for the nucleotide exchange process.

The lack of druggable pockets on the surface of KRAS has been one of the major challenges in developing small molecules that directly target KRAS. The Mg^2+^-free KRAS-GMPPNP structure shows that side chain atoms of A59 can displace Mg^2+^ from the nucleotide binding pocket and result in switch I adopting the state 1 conformation, which is not compatible for effector binding. Small molecules that trigger allosteric conformational changes in residue A59 may lead to irreversible loss of Mg^2+^. Structures of G12C-KRAS mutant protein covalently-bound to small molecules have been used to develop promising leads for directly targeting this oncogenic allele. In some cases, G12C-KRAS covalent binders displace Mg^2+^ from the nucleotide-binding pocket which then facilitates an induced fit binding of the small molecule in the switch II pocket^39^. Thus, small molecules that exploit formation of inducible pockets in the switch regions of KRAS are likely to be successful if they displace Mg^2+^ either directly or by causing conformational change in residue A59 or the interswitch region.

## Materials and Methods

### Characterization of the N-terminus of KRAS4b expressed in HEK293 cells

To investigate the processing at the N-terminus, a variant of human KRAS4b (amino acids 1–171 followed by a His8 tag for purification) was generated. The coding sequences were sub-cloned into mammalian expression vectors driven by the human cytomegalovirus promoter and were transfected into HEK293 cells using the manufacturer’s protocols (Thermo Fisher, Carlsbad, CA). After 72 hours, cell pellets were harvested and lysed by sonication, and proteins were purified using a microscale purification technique with immobilized metal affinity chromatography (IMAC) as previously described^40^. Purified proteins were analyzed by SDS-PAGE. To identify the molecular mass of the purified protein, it was diluted to 0.01 mg/ml and 1 μL was injected onto an Agilent 1200 nanoflow LC system coupled online with an LTQ Orbitrap XL mass spectrometer. ESI-MS spectrum of the protein was acquired in profile mode at 60000 resolution and processed using the Xtract function in Thermo Xcalibur Qual Browser at S/N of 10 to generate its molecular weight information.

### Cloning, expression, and purification of recombinant KRAS proteins

Gateway Entry clones for *E. coli* produced KRAS4b variants and RAF1-RBD (52–131) were generated by standard cloning methods and incorporate an upstream tobacco etch virus (TEV) protease cleavage site (ENLYFQG) followed by the appropriate KRAS or RAF1 sequences. Sequence validated Entry clones were sub-cloned into pDest-566, a Gateway Destination vector containing a His6 and maltose-binding protein (MBP) tag to produce the final *E. coli* expression clones^41^. The BL21 STAR (rne131) *E. coli* strain containing the DE3 lysogen and rare tRNAs (pRare plasmid CmR) was transformed with the expression plasmid (His6-MBP-TEV-protein of interest, AmpR). The N-acetylated KRAS clone was generated using an insect codon optimized KRAS4b construct containing amino acids 1–169 (ATUM Bio, Inc.) which was introduced into a Gateway Entry clone and subsequently subcloned into a pFastBac style native expression vector (pDest-8, Thermo Fisher). Bacmid DNA was generated using the Bac-to-Bac system (Thermo Fisher) using the manufacturer’s instructions, and baculovirus was generated as previously outlined^42^. The expression and purification of KRAS variants and RAF1-RBD described in this study were carried out using the procedure described previously^42^. Briefly, the expressed proteins of the form His6-MBP-TEV- protein of interest were purified from clarified lysates by IMAC, treated with His6-TEV protease to release the target protein (of the form Gly- or Gly-Gly-protein of interest), and the target protein separated from other components of the TEV protease reaction by the second round of IMAC. The N-acetylated KRAS (2–169) was purified from clarified lysate by ion-exchange column chromatography using a Q Sepharose Fast Flow (GE Healthcare). Positive fractions were pooled, the pools concentrated to an appropriate volume for injection onto a 26/60 Superdex S-75 (GE Healthcare) column equilibrated and run in 20 mM HEPES, pH 7.3, 150 mM NaCl, 2 mM MgCl_2_, and 1 mM TCEP. The peak fractions containing pure protein were pooled, flash-frozen in liquid nitrogen and stored at −80 °C.

### Nucleotide exchange

Purified KRAS proteins are bound to GDP. To crystallize KRAS bound to GTP analog, GMPPNP, we carried out nucleotide exchange to replace GDP by GMPPNP. Nucleotide exchange was performed first by exchanging KRAS into a buffer containing 25 mM Tris pH 8.0, 0.1 mM ZnCl_2_, and 200 mM AmSO_4_. This was followed by addition of GMPPNP (molar ratio of 10:1 GMPPNP:KRAS) and alkaline phosphatase beads (3 U per mg of KRAS). The reaction mixture was then gently rotated for 3 hours at room temperature. Protein and alkaline phosphatase beads were separated by centrifugation and supernatant was passed through a 0.22-micron centrifugal filter to remove any residual beads. This was followed by addition of 20 mM MgCl_2_ and an additional amount of GMPPNP (molar ratio of 5:1 GMPPNP: KRAS) and incubation at room temperature for 1 hour. Nucleotide exchanged protein was then desalted and exchanged to a final buffer containing 20 mM HEPES (pH 7.4), 150 mM NaCl, 5 mM MgCl_2_, and 1 mM TCEP. The protein was then concentrated, flash frozen, and stored at −80 °C until it was used for crystallization.

### Crystallization and data collection

Crystallization screenings were carried out using the sitting-drop vapor diffusion method by mixing the nucleotide-bound KRAS with an equal volume of reservoir solution. Crystallization hits from initial screens were optimized by systematically varying the pH and individual component concentrations. Conditions that yielded crystals are summarized in Supplementary Table 1. Crystals were harvested for data collection and cryoprotected with a 25% (v/v) solution of ethylene glycol or glycerol mixed with crystallization solution before being flash-cooled in liquid nitrogen. Diffraction data sets were collected on 21-ID-F/G and 24-ID-C/E beamlines at the Advanced Photon Source (APS), Argonne National Laboratory. Crystallographic datasets were integrated and scaled using the XDS^43^. The crystal parameters and the data collection statistics are summarized in Supplementary Table 2.

### Structure determination and analysis

KRAS structures were solved by molecular replacement using the program Phaser as implemented in the Phenix/CCP4 suite of programs with a protein-only version of PDB entries 3GFT, 4OBE or 4LPK as a search model^44–46^. The initial solution was refined using Refmac5 and the resulting *Fo-Fc* map showed clear electron density for either the GDP or GMPPNP. The model was further improved using iterative cycles of manual model building in COOT^47^ and refinement using Phenix.refine^44^. Placement of ligands was followed by identification of potential sites of solvent molecules by the automatic water-picking algorithm in COOT and Phenix.refine. The positions of these automatically picked waters were checked manually during model building. The alternative conformations of amino acids were added using COOT during final rounds of refinements. Refinement statistics for all the crystal structures are summarized in Supplementary Table 2. Secondary structural elements were assigned using DSSP (http://swift.cmbi.ru.nl/gv/dssp/). Figures were generated with PyMOL (Schrödinger, LLC) and surface electrostatics were calculated with APBS^48^. Crystallographic and structural analysis software support is provided by SBGrid consortium^49^.

### ^1^H-^15^N HSQC NMR experiments

NMR data were collected on an Agilent 800 MHz spectrometer, processed with NMRPIPE^50^ and analyzed via CCPN Analysis^51^ or NMRFAM-SPARKY^52^. ^1^H-^15^N HSQC data were collected on 0.2 mM of GDP-bound ^15^N KRAS (1–169) and ^15^N KRAS (2–169) in a buffer containing 20 mM HEPES (pH 7.2), 150 mM NaCl, 1 mM MgCl_2_ and 1 mM TCEP-HCL at 25 °C. Proton, carbon, and nitrogen NMR chemical shifts for KRAS4b are reported here^53^.

### Circular dichroism measurements

Protein samples of KRAS (1–169)-GDP and KRAS (2–169)-GDP were buffer-exchanged into a buffer consisting of 10 mM HEPES (pH 7.4), 150 mM NaCl, and 1 mM TCEP. The CD measurements were taken on a Chirascan Plus instrument (Applied Photophysics, UK) at a protein concentration of 0.1 mg/ml. The spectra were acquired at room temperature between a wavelength of 200–260 nm using a 200 μl sample in a cell with 1 mm pathlength, with sampling points every 1 nm.

### Intrinsic GTP hydrolysis assay

The intrinsic GTPase hydrolysis assay for KRAS(1–169) and KRAS(2–169) was performed as described previously^18^. Specifically, 2 μM KRAS with 2 μCi γ^32^P GTP in 50 mM HEPES (pH 7.3), 50 mM NaCl, 1 mM TCEP and 10 mM EDTA was incubated at 37 °C for 15 minutes to exchange GDP for labeled GTP in a total volume of 100 μl. Unincorporated ^32^P GTP was removed using a desalting column. Approximately 3 μg of each KRAS4b-^32^P-labeled GTP sample was incubated at 37 °C in 300 μl of hydrolysis buffer supplemented with 1 mM MgCl_2_. Aliquots were removed and added to 400 μl of prechilled stop buffer that consisted of 5% (w/v) activated charcoal, 0.2 M HCl, 1 mM NaH_2_PO_4_ and 20% (v/v) ethanol. After centrifugation soluble ^32^P counts were measured by scintillation counting. Observed counts at each time point were converted to a percentage of total counts. Percentage results were modeled with an exponential function; rates and associated variation were extracted using MATLAB’s nonlinear regression system.

### Isothermal titration calorimetry measurements

Binding affinities of GMPPNP-bound KRAS (2–169) and KRAS (1–169) with RAF1-RBD were measured using isothermal titration calorimetry (ITC). Protein samples were prepared by extensively dialyzing them in a buffer (filtered and degassed) containing 20 mM HEPES (pH 7.3), 150 mM NaCl, 5 mM MgCl_2_ and 1 mM TCEP. For the ITC experiment, 60 μM of KRAS and 600 μM of RAF1-RBD were placed in the cell and syringe, respectively. ITC experiments were performed in a MicroCal PEAQ-ITC (Malvern) at 25 °C using 19 injections of 2.2 μl injected at 150-s intervals. Data analysis was performed based on a binding model containing “one set of sites” using a nonlinear least squares algorithm incorporated in the MicroCal PEAQ-ITC analysis software (Malvern).

### Intrinsic and SOS-mediated nucleotide exchange assay

Wild-type KRAS with and without the iMet (1–169 and 2–169) were exchanged to mant-GDP using the protocol described previously^54^. The samples were divided into two aliquots and desalted, one into buffer with MgCl_2_, and one into buffer without MgCl_2_. The first set was carried out in a 3 mL cuvette containing reaction buffer (40 mM HEPES, 150 mM NaCl, 1 mM TCEP and 5% glycerol) mixed with 1.5 μM of KRAS labelled with mant-GDP, and 3.5 mM of GDP in the presence and absence of 2.5 μM SOS. The second set was carried out in a reaction buffer that included 4 mM MgCl_2_. Nucleotide exchange rates were monitored on a Horiba Jobin Yvon Fluorolog Fluorometer at 25 °C for 120 minutes (with measurements taken every 15 s), with 355 nm excitation and 448 nm emission. Data were fit to a single exponential decay curve for quantification of intrinsic and SOS-mediated nucleotide exchange rates.

### PDB accession numbers

The atomic coordinates and structure factors of the various complexes have been deposited in the Protein Data Bank under the following ID codes: 6P0Z: Mg^2+^-bound N-acetylated KRAS (2–169)-GDP; 6M9W: Mg^2+^-free KRAS (2–169)-GDP; 6MBU: Mg^2+^-bound KRAS (1–169)-GDP (P3, crystal form I); 6MBT: Mg^2+^-bound KRAS (1–169)-GDP (C2, crystal form II); 6MBQ: Mg^2+^-free KRAS (2–166)-GMPPNP.

## Supplementary information

Supplementary Information
