# Supplementary material for: Structures of N-terminally processed KRAS provide insight into the role of N-acetylation
Source: Sci Rep. 2019 Jul 19;9:10512. doi: 10.1038/s41598-019-46846-w (PMC6642148; doi:10.1038/s41598-019-46846-w)
Supplement: Supplementary file 1 — Supplementary Information [file 41598_2019_46846_MOESM1_ESM.pdf]

Structures of N-terminally processed KRAS provide insight into the role of N-acetylation

Srisathiyanarayanan Dharmaiah[‡], Timothy H. Tran[‡], Simon Messing[‡], Constance Agamasu[‡], William K. Gillette[‡], Wupeng Yan[‡], Timothy Waybright[‡], Patrick Alexander[‡], Dominic Esposito[‡], Dwight V. Nissley[‡], Frank McCormick[‡, §], Andrew G. Stephen[‡], and Dhirendra K. Simanshu[‡,*]

[‡]NCI RAS Initiative, Cancer Research Technology Program, Frederick National Laboratory for Cancer Research, Leidos Biomedical Research, Inc., Frederick, MD 21701, [§]Diller Family Comprehensive Cancer Center, University of California, San Francisco, CA 94158

Supplementary Information

- Supplementary Table 1- 2
- Supplementary Figures 1- 8

Supplementary Table 1: Crystallization conditions for KRAS structures described in this study.

KRAS structures	Crystallization condition
Mg^{2+}-free KRAS (2-169)-GDP	0.056 M $NaPO_4.H_2O$, 1.344 M K_2PO_4, pH 8.2
Mg^{2+}-bound KRAS (1-169)-GDP (P3)	0.2 M $Mg^{2+}Cl_2$, 0.1 M TRIS HCl pH 8.5, 30% PEG 4K
Mg^{2+}-bound KRAS (1-169)-GDP (C2)	6% 2-Propanol, 0.1 M $NaOAc_3H_2O$ pH 4.5, 26% PEG MME 550
Mg^{2+}-free KRAS (2-166)-GMPPNP	200 mM Ammonium Fluoride, 20% PEG 3350
Mg^{2+} bound N-acetylated KRAS (2-169)-GDP	10% w/v PEG 20 000, 20% v/v PEG MME 550, 0.1 M MES/imidazole pH 6.5, 0.02 M of each amino acid (L-Na-Glutamate; Alanine (racemic); Glycine; Lysine HCl (racemic); Serine (racemic))

Supplementary Table 2. Data collection and refinement statistics.

	Mg^{2+}-free KRAS (2-169)-GDP	Mg^{2+}-bound KRAS (1-169)-GDP (P3)	Mg^{2+}-bound KRAS (1-169)-GDP (C2)	Mg^{2+}-free KRAS (2-166)-GMPPNP	Mg^{2+}-bound N-acetylated KRAS (2-169)-GDP
**Data Collection**					
**Resolution range (Å)**	42.95 - 1.5 (1.55 - 1.5)	41.92 - 1.45 (1.50 - 1.45)	19.47 - 1.45 (1.5 - 1.45)	35.45 - 1.35 (1.39 - 1.35)	37.23-1.01 (1.05-1.01)
**Space group**	P 3 2_1	P 3	C 1 2 1	P 2_1 2_1 2_1	C 1 2 1
**Unit cell** a, b, c (Å) α, ß, γ (°)	77.86, 77.86, 55.73 90, 90, 120	84.18, 84.18, 41.92 90 90 120	66.29, 41.55, 115.38 90, 105.05, 90	36.02, 37.91, 99.99 90, 90, 90	65.19, 41.57, 115.41 90 104.58 90
**Total reflections**	226355 (22073)	161902 (16251)	185909 (18425)	208763 (19052)	865278 (25971)
**Unique reflections**	31532 (3103)	58439 (5924)	52619 (5182)	30423 (2915)	148832 (11112)
**Multiplicity**	7.2 (7.1)	2.8 (2.8)	3.5 (3.6)	6.9 (6.5)	5.8 (2.3)
**Completeness (%)**	99.77 (99.65)	99.29 (100.0)	96.94 (96.32)	98.37 (95.82)	95.28 (71.39)
**Mean I/sigma(I)**	18.71 (2.29)	8.73 (1.88)	11.72 (2.27)	24.53 (6.84)	14.84 (1.02)
**Wilson B-factor ($Å^2$)**	17.25	13.40	14.52	8.99	9.68
**R-merge**	0.059 (0.678)	0.070 (0.46)	0.056 (0.510)	0.0481 (0.255)	0.0559 (0.6361)
**R-meas**	0.065 (0.731)	0.087 (0.572)	0.066 (0.602)	0.0521 (0.277)	0.0609 (0.7961)
**CC1/2**	0.999 (0.847)	0.993 (0.724)	0.998 (0.858)	1 (0.99)	1 (0.904)
**Refinement**					
**Reflections used in refinement**	31532 (3101)	58509 (5924)	52618 (5176)	30423 (2913)	148832 (11100)
**Reflections used for R-free**	2006 (202)	2964 (316)	2007 (200)	1519 (142)	1996 (153)
**R-work**	0.144 (0.214)	0.134 (0.187)	0.177 (0.245)	0.135 (0.160)	0.1445 (0.3575)
**R-free**	0.186 (0.289)	0.182 (0.240)	0.216 (0.299)	0.184 (0.209)	0.1663 (0.3748)
**CC (work)**	0.97 (0.93)	0.59 (0.16)	0.97 (0.90)	0.97 (0.94)	0.974 (0.797)
**CC (free)**	0.96 (0.89)	0.57 (0.19)	0.96 (0.85)	0.96 (0.90)	0.970 (0.709)
**Number of non-H atoms**	1729	3083	3230	1644	3395
**macromolecules**	1469	2705	2824	1422	2859
**ligands**	28	79	58	37	71
**solvent**	232	299	348	185	465
**Protein residues**	168	328	340	169	334
**RMS (bonds)**	0.003	0.003	0.005	0.009	0.007
**RMS (angles)**	0.68	0.67	0.83	1.05	1.06
**Ramachandran favoured (%)**	98.80	97.83	99.11	98.80	99.09
**Ramachandran allowed (%)**	1.20	2.17	0.60	1.20	0.91
**Ramachandran outliers (%)**	0.00	0.00	0.30	0.00	0.00
**Average B-factor ($Å^2$)**	25.05	22.35	16.83	12.58	14.73
**macromolecules**	22.65	21.09	15.46	11.21	12.71
**ligands**	17.51	17.22	14.47	7.68	13.51
**solvent**	41.18	35.15	28.34	24.15	27.34

Statistics for the highest-resolution shell are shown in parentheses.

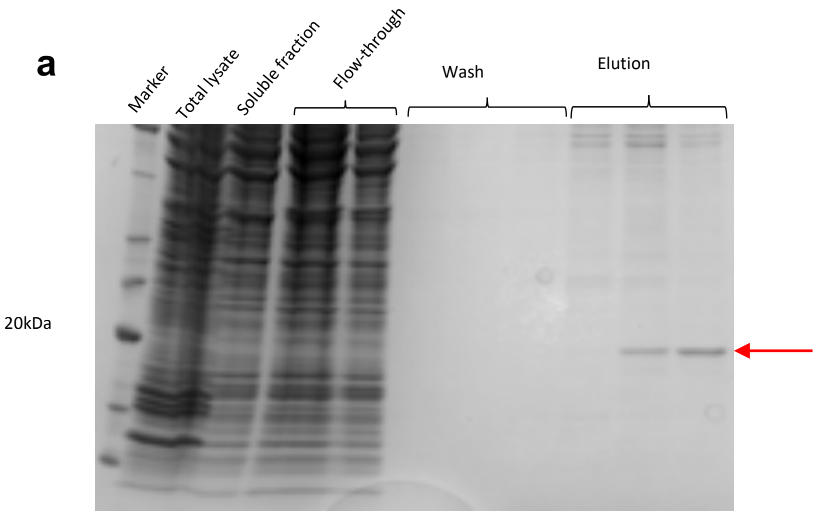

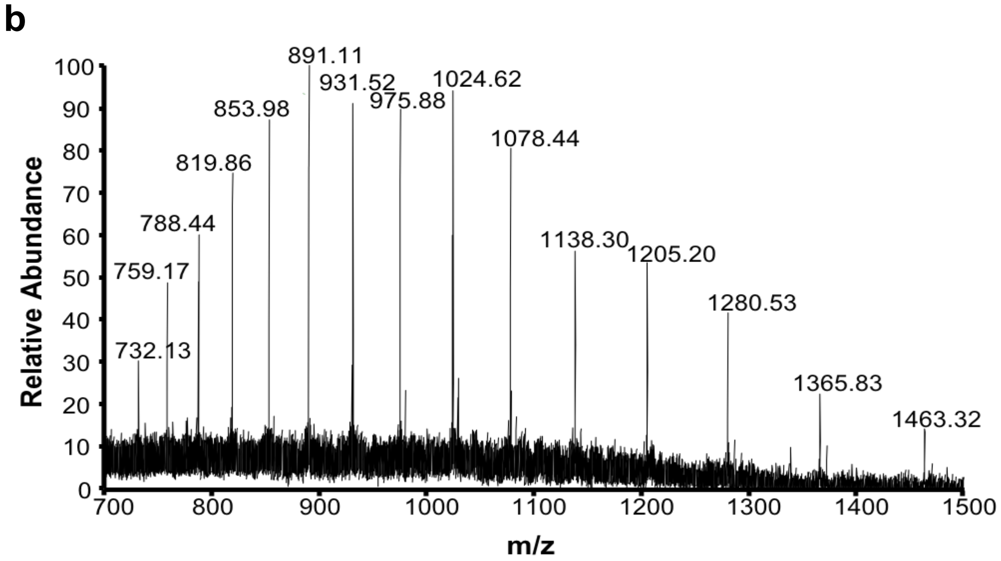

Supplementary Figure 1: Mass spectroscopic characterization of KRAS4b expressed in HEK293 cells. (**a**) SDS-PAGE analysis of human KRAS4b (1-171 with a C-terminal His8 tag) expressed in HEK293 cells and purified using immobilized metal affinity chromatography (IMAC). Red arrow denotes purified KRAS4b. (**b**) The electrospray ionization mass spectrometry (ESI-MS) spectrum of the purified protein is shown. The deconvoluted spectrum is shown in Figure 1.

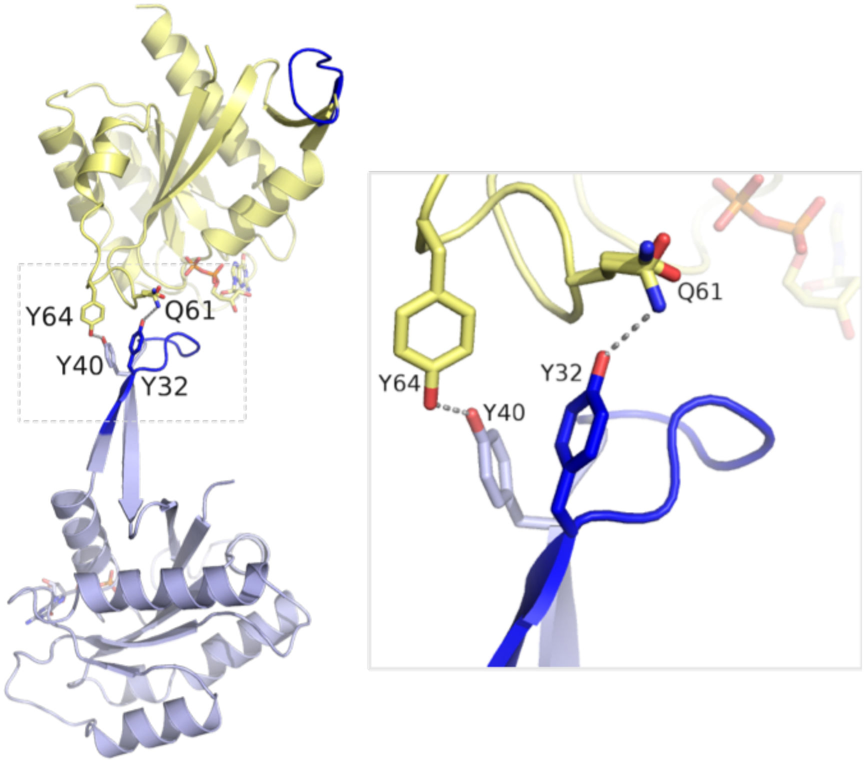

Supplementary Figure 2: Interaction formed by switch I residues in the Mg^{2+}-free KRAS-GDP structure with the residues from the symmetry related KRAS molecule. Residues Y32 and Y40 of Mg^{2+}-free KRAS-GDP (shown in light blue with switch I region highlighted in dark blue) present in the asymmetric unit form H-bonds with Q61 and Y64, respectively, from the symmetry related molecule. Enlarged view of this interaction (shown in box) is shown in the right panel.

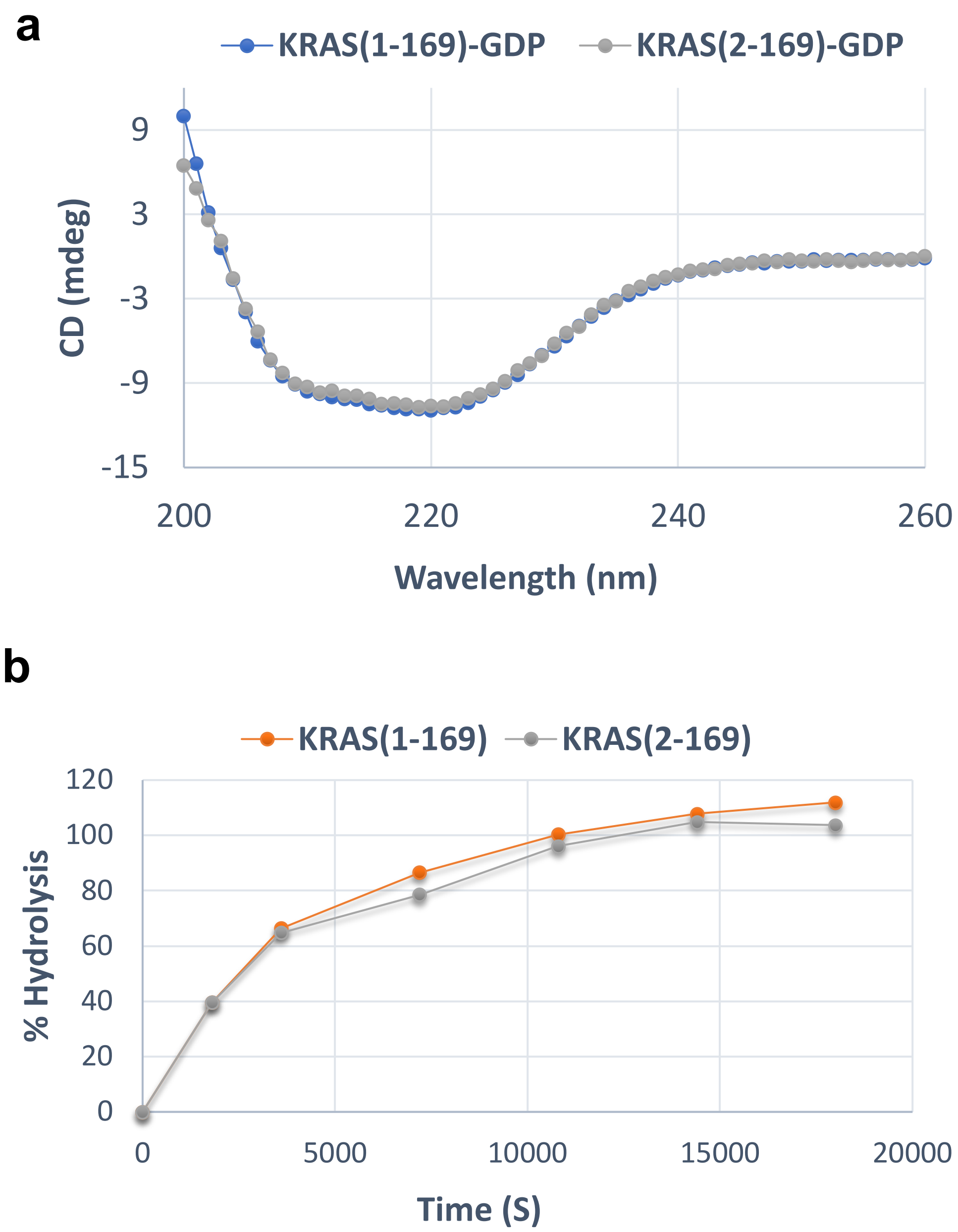

Supplementary Figure 3: Comparison of secondary structural content and rate of intrinsic GTP hydrolysis for KRAS (1-169) and KRAS (2-169). (**a**) Circular dichroism spectra for GDP-bound KRAS (1-169) and KRAS (2-169). (**b**) Plot showing rate of intrinsic GTP hydrolysis for KRAS (1-169) and KRAS (2-169).

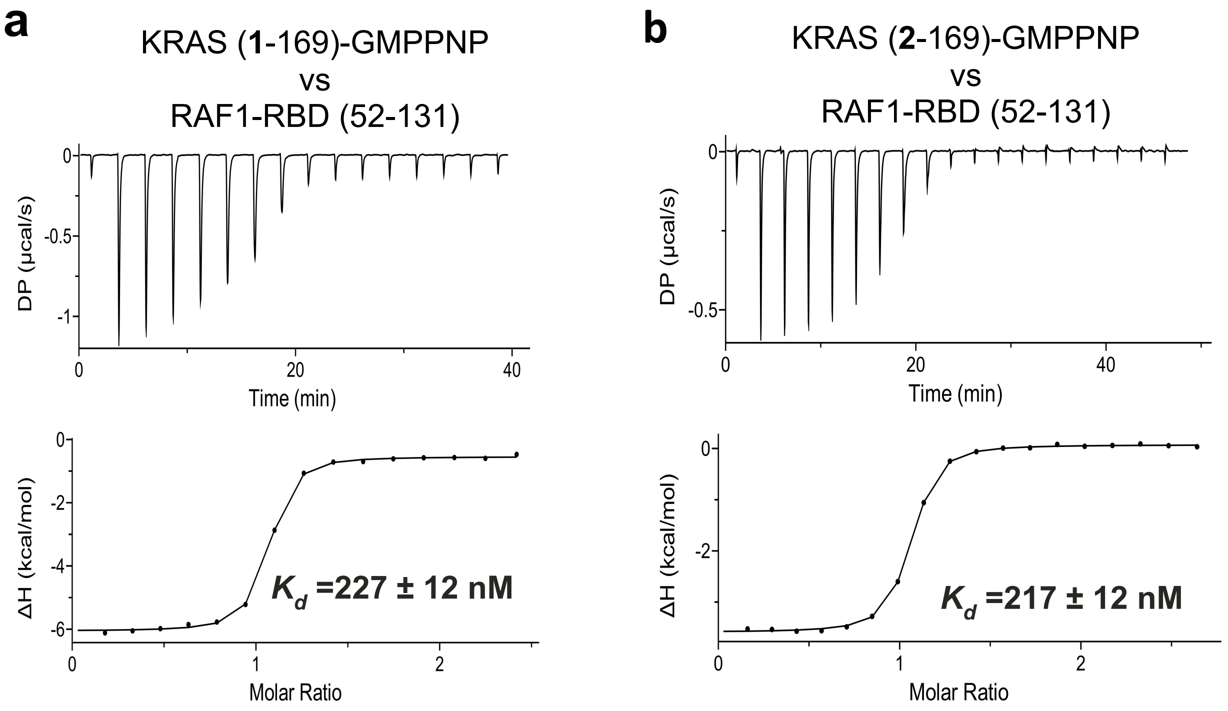

Supplementary Figure 4. Binding affinity of GMPPNP-bound KRAS (1-169) and KRAS (2-169) with RAS-binding domain (RBD) of RAF1. Isothermal titration calorimetry (ITC) experiments to measure the dissociation constant between (**a**) KRAS (1-169)-GMPPNP and RAF1-RBD and between (**b**) KRAS (2-169)-GMPPNP and RAF1-RBD. The DP is a measured differential power between the reference cell and the sample cell.

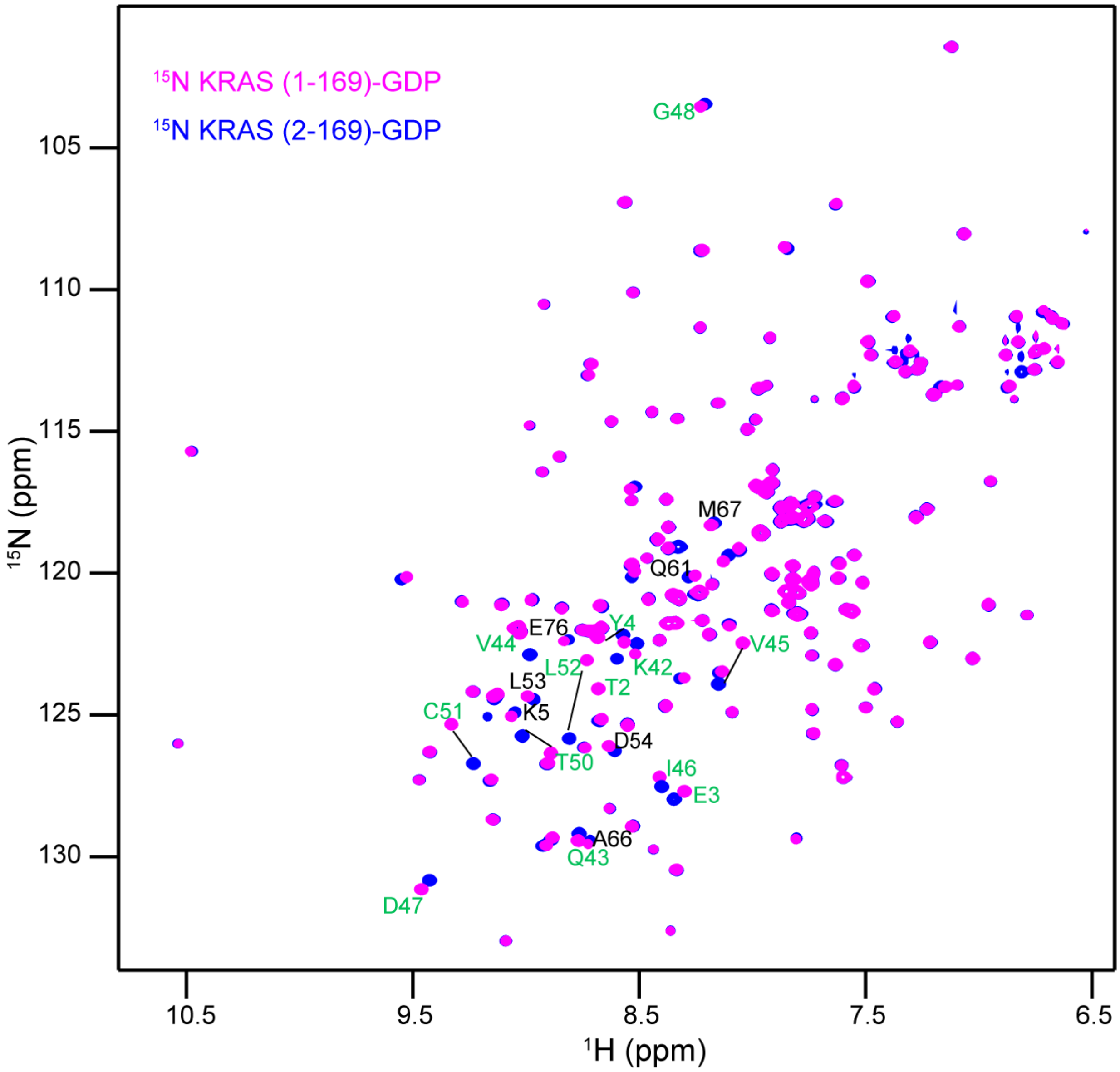

Supplementary Figure 5. Comparison of HSQC spectra obtained for GDP-bound KRAS (2-169) and KRAS (1-169). 2D ^{1}H-^{15}N HSQC spectra obtained for ^{15}N-labeled KRAS (1-169) (magenta) and free ^{15}N labeled KRAS (2-169) (blue). Amide signals that exhibited substantial chemical shift differences are labeled in green.

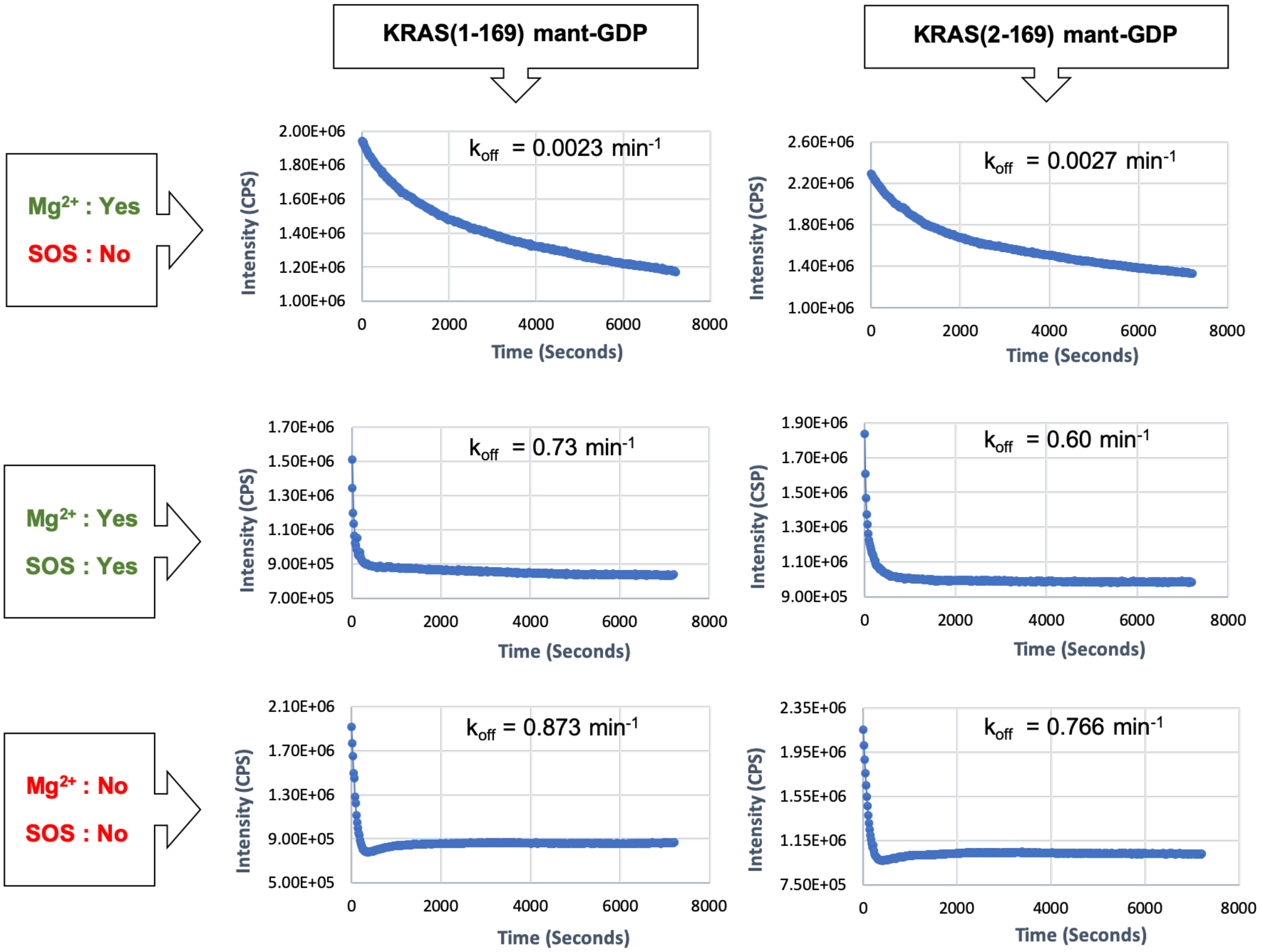

Supplementary Figure 6: Intrinsic and SOS-mediated nucleotide exchange rate for KRAS proteins in the presence and absence of initiator methionine. In the presence of Mg^{2+} in the reaction mixture, both KRAS proteins (1-169 and 2-169) showed similar rates for intrinsic and SOS-mediated nucleotide exchange. However, in the absence of Mg^{2+} in the reaction mixture, both KRAS proteins showed intrinsic exchange rates higher than the SOS-mediated exchange rate obtained in the presence of Mg^{2+}. When SOS was added to this Mg^{2+}-free reaction mixture, the reaction occurred so rapidly that the majority of the nucleotide was displaced before the first measurement was made (data not shown).

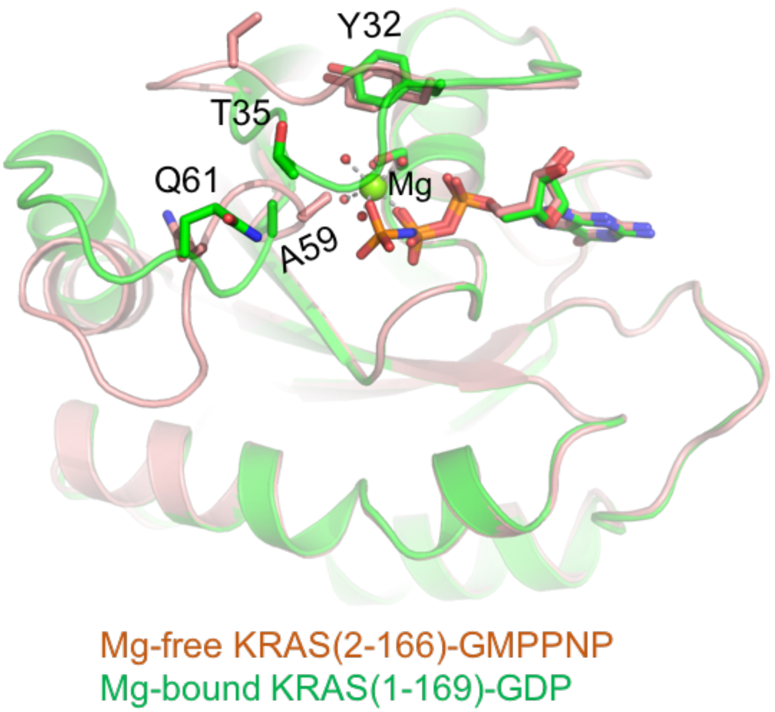

Supplementary Figure 7: Structural superposition of Mg^{2+}-free KRAS (2-166)-GMPPNP with Mg^{2+}-bound KRAS (1-169)-GDP structures. KRAS is shown in ribbon representation, GDP and sidechain atoms of KRAS are shown in stick representation.

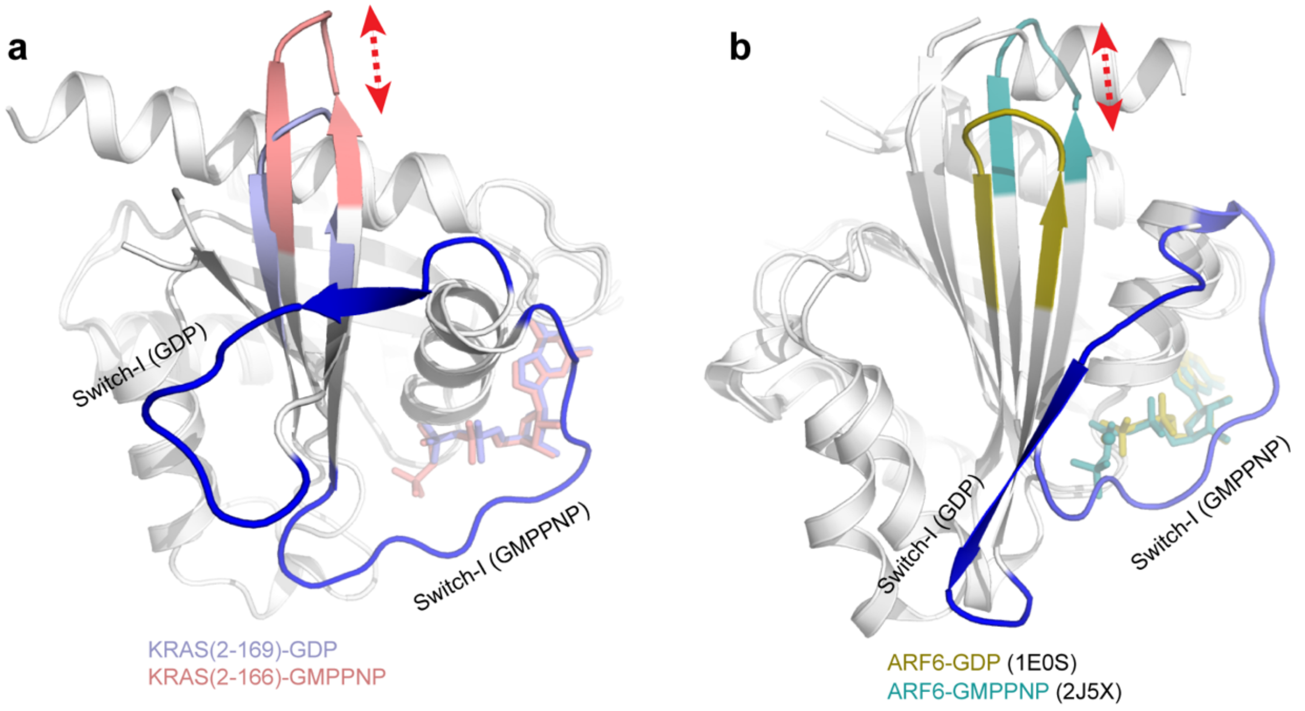

Supplementary Figure 8: Mg^{2+}-free KRAS structures resemble conformational changes seen previously in Mg^{2+}-bound ARF6 structures. Structural superposition of (**a**) Mg^{2+}-free GDP/GMPPNP-bound KRAS structures and (**b**) Mg^{2+}/GDP/GMPPNP-bound ARF6 structures showing presence of additional β-strand in the switch I region as well as conformational changes in the turn between β2 and β3 strands.
